# HIF-1*α* RNAi Combined with Asparagus Polysaccharide Exerts an Antiangiogenesis Effect on Hepatocellular Carcinoma In Vitro and In Vivo

**DOI:** 10.1155/2021/9987383

**Published:** 2021-07-23

**Authors:** Tingting Zhu, Ziwei Cheng, Xiaolin Peng, Dongwei Xing, Minguang Zhang

**Affiliations:** Shanghai Municipal Hospital of Traditional Chinese Medicine, Shanghai University of Traditional Chinese Medicine, Shanghai 200071, China

## Abstract

**Background:**

Hepatocellular carcinoma (HCC) is the main form of primary liver cancer and is one of the most prevalent and life-threatening malignancies globally. Hypoxia activates hypoxia-inducible factor-1*α* (HIF-1*α*), which is the key factor in promoting angiogenesis in HCC. Currently, there are few studies on the effects of HIF-1*α*-targeted gene therapy combined with traditional Chinese herbal extracts.

**Objective:**

We investigated the effects of HIF-1*α* RNA interference (RNAi) combined with asparagus polysaccharide (ASP) on HCC in vitro and in vivo.

**Methods:**

CCK-8, wound-healing, transwell, and human umbilical vein endothelial cell tube formation assays were performed to evaluate the proliferation, migration, invasion, and angiogenesis of HCC cells in vitro. In addition, western blotting, qPCR, and immunohistochemistry were performed to detect the expression of HIF-1*α*, vascular endothelial growth factor, AKT, p-AKT, ERK, p-ERK, and CD34 in HCC cells.

**Results:**

The combination of HIF-1*α* RNAi and ASP significantly inhibited the proliferation, migration, invasion, and angiogenesis of SK-Hep1 and Hep-3B cells compared with the use of HIF-1*α* RNAi or ASP alone. In addition, this combined treatment was shown to exert these effects by regulating the PI3K and MAPK signaling pathways. These results were observed both in vitro and in vivo.

**Conclusion:**

Our study indicates that HIF-1*α* RNAi combined with ASP inhibits angiogenesis in HCC via the PI3K and MAPK signaling pathways. Thus, we suggest that this combination may be an effective method for the comprehensive treatment of HCC, which may provide new ideas for the treatment of other malignant tumors.

## 1. Introduction

Hepatocellular carcinoma (HCC) is a common malignancy of the digestive system. Globally, the number of new cases reaches 841,000 every year, making it the sixth most prevalent malignant tumor, and the number of deaths is 782,000 per year, ranking second among malignant tumors. China has a high incidence of HCC, and more than half of all HCC cases worldwide occur in this country [1–3]. Early-stage HCC can be treated curatively by local ablation, surgical resection, or liver transplantation, but only 40% of patients with HCC are diagnosed at an early stage [4, 5].

HCC is a solid tumor rich in blood vessels, and angiogenesis plays a crucial role in its occurrence, development, recurrence, and metastasis; thus, intrahepatic metastasis can occur early with poor prognosis. Hypoxia-inducible factor-1*α* (HIF-1*α*) is the most essential transcriptional regulator of hypoxia response. It has been well demonstrated that HIF-1*α* extensively regulates hypoxia gene expression and aggressive phenotypes of cancer cells, leading to metabolic changes, increased survival, invasion, migration, angiogenesis, and other related signal transduction [6, 7]. Recent studies have shown that overexpression of HIF-1*α* is always detected in HCC and is associated with poor clinical outcomes [8–10]. RNA interference (RNAi), as a promising gene therapy method, has higher silencing efficiency and stability than traditional gene therapy and can target and interfere with pathogenic genes in a sequence-specific manner, providing more accurate and personalized treatment for a variety of life-threatening diseases [11, 12]. Especially for malignant tumors, RNAi technology has significant advantages in controlling tumor growth, inhibiting tumor angiogenesis-related factors, and reducing tumor drug resistance [13]. The occurrence and development of many malignant tumors, including liver cancer, is the result of the accumulation of gene mutations and regulation of gene networks formed by the interaction of these mutated genes. Therefore, inhibiting the expression of these genes using RNAi technology may prevent the occurrence and development of HCC. With the deepening of research on the molecular mechanisms of HCC, an increasing number of genes have been found to be associated with this disease, which makes them targetable using RNAi technology. Hence, the application of gene interference technology to suppress HIF-1*α*-induced signaling pathways may be a novel strategy for HCC treatment.

Using gene targeted therapy, we innovatively explored whether we can combine modern medical technology with traditional Chinese medicine to achieve more effective prevention and treatment of tumors. Asparagus is a traditional Chinese herbal medicine that contains bioactive compounds such as flavonoids, lignans, steroids, and saponins, which are widely used in the treatment of breast cancer, malignant lymphoma, leukemia, and lung cancer [14]. Asparagus polysaccharide (ASP) is the main active component of asparagus and exerts antitumor and proapoptotic effects. In our previous study, we reported the effects of ASP on the growth, migration, invasion, and angiogenesis of HCC cells under hypoxia [15, 16]. Based on the findings of previous studies, we continued to study the auxiliary role of ASPs in RNAi. This combined treatment has a dual targeting effect that may improve the local concentration of tumor drugs and transfection effect, thus increasing the sensitivity to gene therapy and achieving a more satisfactory antitumor effect. The results of this study can provide a theoretical basis for the introduction of gene therapy into clinical practice and a new perspective and breakthrough for the treatment of liver cancer and other tumors.

## 2. Materials and Methods

### 2.1. Cell Lines and Cell Culture

SK-Hep1 and Hep-3B cell lines and human umbilical vein endothelial cells (HUVECs) were purchased from the Type Culture Collection of the Chinese Academy of Sciences (Shanghai, China). Cells were divided into four groups: control group, ASP (24-h exposure)-treated group, HIF-1*α* RNAi-treated group, and HIF-1*α* RNAi and ASP (24-h exposure)-treated group. All cells were maintained in DMEM (HyClone, Logan, USA) supplemented with 10% fetal bovine serum (FBS) (Gibco, South America) and 1% penicillin-streptomycin (Gibco). To establish hypoxia, SK-Hep1 and Hep-3B cells were cultured in a hypoxic incubator (37°C, 5% CO_2_, and 1% O_2_) for 24 h. HUVECs were cultured in a normal oxygen incubator (37°C, 5% CO_2_, and 20% O_2_).

### 2.2. ASP

ASP was purchased from Yuanye (Shanghai, China). DMEM was used to dilute ASP to 200 mg/mL, and ASP was dissolved overnight on a shaking table at 4°C. The next day, the supernatant liquid was centrifuged at 1000 rpm for 5 min, and the supernatant was filtered using 0.45 and 0.22 *μ*m filters and stored at −20°C for long-term use.

### 2.3. Lentivirus and Plasmid Transfection

The adenovirus vector was amplified and packaged by Genechem (Shanghai, China). According to the manufacturer's instructions, SK-Hep1 and Hep-3B cells were cultured in a 6-well plate 1 d before the experiment. Then, shHIF1*α* lentiviruses were transfected into SK-Hep1 and Hep-3B cells. These cells were cultured for 8–12 h to observe their state, and the medium was replaced with a fresh and complete medium. After infection for 72 h, cells were observed under a fluorescence microscope and stably transfected cell lines were obtained after culturing in the medium containing puromycin (2–3 g/mL) for 2 weeks. The shRNA targeting sequences for HIF-1*α* were as follows: 5′-TGACAAGCCACCTGAGGAGA-3′ and 5′-ACACGCGGAGAAGAGAAGGA-3′.

### 2.4. CCK-8 Cell Viability Assay

The CCK-8 assay was used to assess cell viability. Cells in 6-well plates were trypsinized to obtain a cell suspension and cultured at 1 × 10^4^ cells/well in a 96-well plate. Each group was cultured in three wells, with another three wells (100 *μ*L DMEM) serving as a blank control, and the 96-well plate was incubated in a hypoxic incubator (37°C, 5% CO_2_, 1% O_2_) for 24 h. Then, the medium was aspirated and discarded, and 10 *μ*L CCK-8 proliferation reagent (Dojindo, Tokyo, Japan) and 90 *μ*L DMEM were added to each well. The absorbance (OD value) of each group was measured at 450 nm using an enzyme standard instrument (Thermo Fisher Scientific, Waltham, MA, USA). Cell viability was calculated as follows: cell activity inhibition rate (%) = ((average OD value of the control group − average OD value of the experimental group)/(average OD value of the control group − average OD value of the blank group)) × 100%.

### 2.5. Wound-Healing Assay

We performed a wound-healing assay to assess the cell migration ability. Cells in the logarithmic growth phase were cultured in a 6-well plate, and the next day, the cell density reached approximately 80%–90%. Two days after culture, the medium was aspirated, and a 20 *μ*L white pipette tip was drawn along a straight line in the center of the wells to form a single scratch. PBS (KeyGEN BioTECH, Jiangsu, China) was used to gently wash off the shed cells, leaving a clean scratch. Next, 2 mL DMEM containing ASP (10 mg/mL) was added to the wells, and 2 mL DMEM was added to the control group and HIF-1*α* RNAi-treated group followed by incubation for 24 h. Scratched wells were photographed under an inverted microscope at 0 h and 24 h (migration rate = scratch area at 0 h/scratch area at 24 h).

### 2.6. Transwell Invasion Assay

Cell invasion ability was assessed using transwell assays. Matrigel (10.5 mg/mL; BD Biosciences, San Jose, CA, USA) diluted to 1/4 in DMEM was spread in the upper compartment of the transwell (100 *μ*L/well) and placed in an incubator for coagulation. Meanwhile, 500 *μ*L DMEM (20% FBS) was added to the lower chambers of the transwell. Then, 1 × 10^5^ cells from each group were added to the upper compartment, incubated for 48 h, fixed with 95% ethanol, stained with crystal violet, and photographed under a light microscope, and the number of invading cells was calculated.

### 2.7. HUVEC Tube Formation Assay

Supernatants were collected from the four groups of cells cultured for 24 h, centrifuged, and stored at −20°C for subsequent use. HUVECs were resuspended in the supernatant at 2.5 × 10^4^ cells/100 *μ*L. Next, 50 *μ*L of Matrigel was added to each well. After incubation at 37°C for 30 min, 100 *μ*L of resuspended HUVECs was added to the wells and incubated at 37°C for 4–6 h. Cells were stained with calcein (Invitrogen, Carlsbad, CA, USA) for 30 min at 37°C. The formation of capillary-like structures was photographed, and Image J software (Image J 1.4; National Institutes of Health, Bethesda, USA) was used to analyze the tube number.

### 2.8. RNA Purification and qPCR Assay

Total RNA was extracted from SK-Hep1 and Hep-3B cells using TRIzol reagent (Thermo Fisher Scientific). A qRT-PCR kit (Takara, Shiga, Japan) and 0.5 *μ*g total RNA were used to perform qRT-PCR. All primers were synthesized by Sangon Biotech (Shanghai, China): GAPDH forward, 5′-CAGGAGGCATTGCTGATGAT-3′, and reverse, 5′-GAAGGCTGGGGCTCATTT-3′; HIF-1*α* forward, 5′-TGACAAGCCACCTGAGGAGA-3′, and reverse, 5′-ACACGCGGAGAAGAGAAGGA-3′; VEGF forward, 5′-TACCTCCACCATGCCAAGTG-3′, and reverse, 5′-GGTCTCGATTGGATGGCAGT-3′.

### 2.9. Western Blotting Assay

Cells at a density of 3 × 10^5^ cells/well were incubated overnight in a 6-well plate. After incubation with different drugs for 24 h, RIPA lysis buffer containing a protease inhibitor and phenylmethylsulfonyl fluoride (Beyotime BioTECH, Shanghai, China) was used for cell lysis on ice for 30 min. Protein concentration was determined using a BCA assay kit (Beyotime BioTECH). Protein samples were added to a protein-loading buffer (Beyotime BioTECH), boiled at 100°C for 8 min, separated using polyacrylamide gel electrophoresis (Epizyme, Shanghai, China), and transferred to a polyvinylidene fluoride membrane (Millipore, Massachusetts, USA). The membrane was sealed with a rapid blocking solution (Beyotime BioTECH) for 30 min at room temperature and then incubated with primary antibodies (diluted at 1 : 1000) at 4°C overnight. Antibodies against HIF-1*α*, VEGF, AKT, p-AKT, ERK, and p-ERK were purchased from CST (Boston, USA). Secondary antibodies labeled with horseradish peroxidase (KeyGEN BioTECH) were then incubated (diluted at 1 : 1500) at room temperature for 1 h. Finally, an ECL chromogenic solution (Beyotime BioTECH) was prepared and dropped on the membrane surface, which was exposed and photographed.

### 2.10. Animal Studies

Twenty male nude mice (Balb/c) (SLAC Laboratory Animal, Shanghai, China) aged 5 weeks were subcutaneously inoculated with 100 *μ*L of PBS containing 5 × 10^6^ SK-Hep1 cells. The mice were divided into the following groups (*n* = 5): (1) control group, inoculated with normal SK-Hep1 cells and treated with normal saline after tumor formation; (2) ASP group, inoculated with normal SK-Hep1 cells and treated with 100 mg/kg ASP (by gavage) after tumor formation; (3) HIF-1*α* RNAi group, inoculated with HIF-1*α* RNAi-treated SK-Hep1 cells and treated with normal saline after tumor formation; and (4) HIF-1*α* RNAi + ASP group, inoculated with HIF-1*α* RNAi-treated SK-Hep1 cells and treated with 100 mg/kg ASP (by gavage) after tumor formation. Tumor volume and body weight were measured daily after the inoculation. Then, the tumor tissues were harvested, embedded, fixed, and prepared for immunohistochemical (IHC) staining and western blotting. All animal experiments performed in this study were carried out under specific pathogen-free conditions and conformed to the requirements of the Animal Ethics Committee.

### 2.11. Statistical Analysis

Each experiment was performed in triplicate. Statistical analyses were performed using the GraphPad Prism, version 8.0. Data (GraphPad Software, San Diego, CA, USA) are expressed as the mean ± SEM. Differences between the groups were estimated using one-way analysis of variance (ANOVA). Statistical significance was set at *P* < 0.05.

## 3. Results

### 3.1. HIF-1*α* RNAi Combined with ASP Inhibited HCC Cell Proliferation

**Figure 1:**
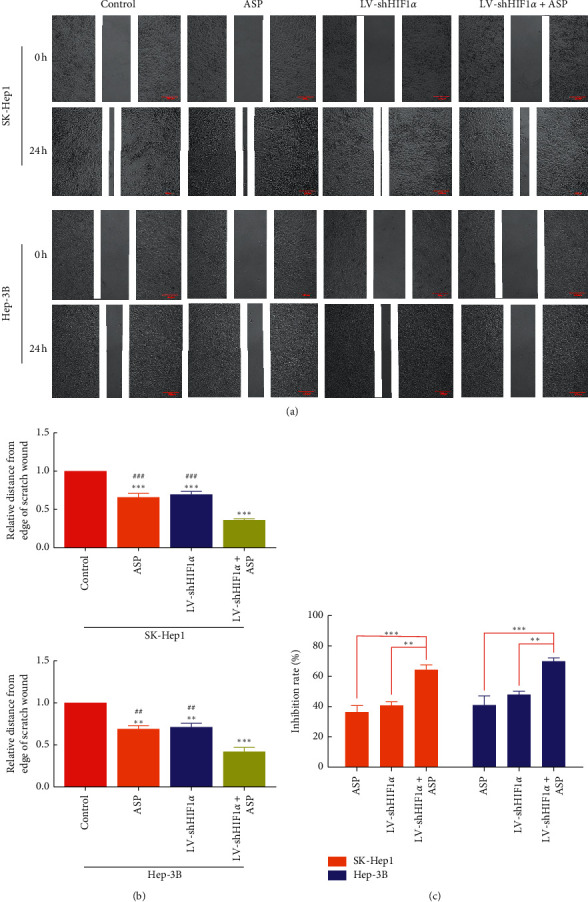
Effect of HIF-1*α* RNAi combined with ASP on the proliferation and migration of SK-Hep1 and Hep-3B cells. (a)-(b) Migration of HCC cells. ^*∗∗*^*P* < 0.01 and ^*∗∗∗*^*P* < 0.001 versus control group. ^##^*P* < 0.01 and ^###^*P* < 0.001 versus LV-shHIF1*α* + ASP group. (c) Proliferation of HCC cells. ^*∗∗*^*P* < 0.01 and ^*∗∗∗*^*P* < 0.001 versus LV-shHIF1*α* + ASP group.

In our previous study [16], we confirmed that different concentrations of ASP inhibited the proliferation of HCC cells. In the present study, based on the IC50 of ASP (12.81 mg/mL for SK-Hep1 and 9.04 mg/mL for Hep-3B), we selected 10 mg/mL ASP for experiments on the combined effect of ASP and HIF-1*α* RNAi on SK-Hep1 and Hep-3B cell proliferation. Compared with the ASP-treated group or HIF-1*α* RNAi-treated group, the proliferation of both cell lines was significantly inhibited by the combination of Asp and HIF-1*α* RNAi. (Figure 1(c)).

### 3.2. HIF-1*α* RNAi Combined with ASP Inhibited the Migration and Invasion of HCC Cells

**Figure 2:**
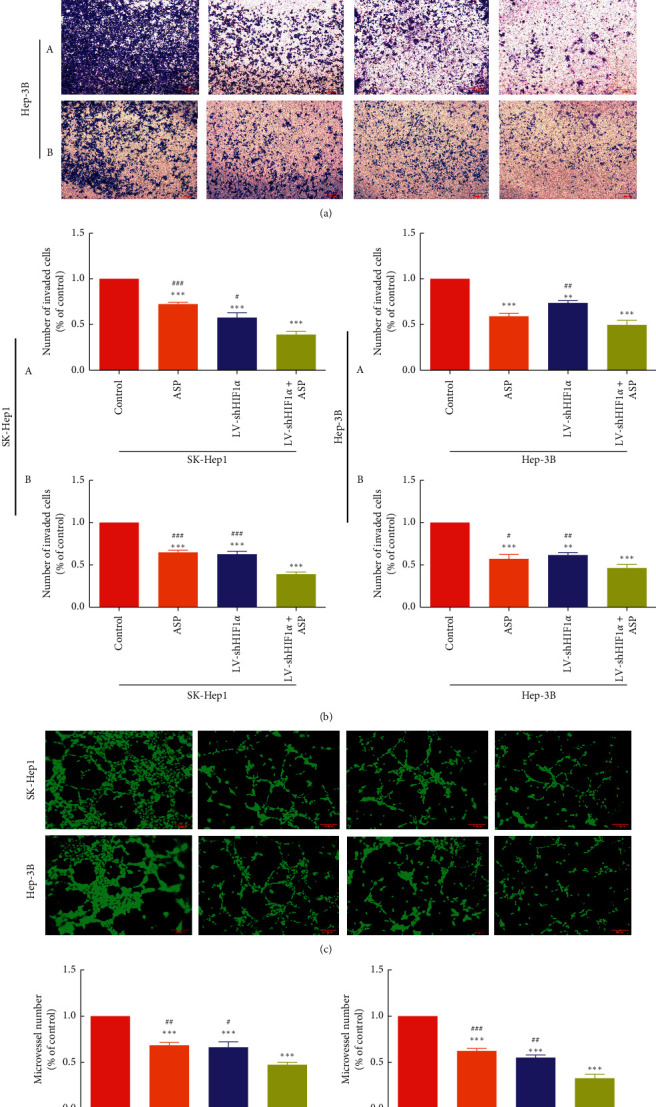
Effect of HIF-1*α* RNAi combined with ASP on the migration and angiogenesis of SK-Hep1 and Hep-3B cells. (a)-(b) Invasion of HCC cells (A. transwell assay; B. transwell invasion assay with Matrigel). ^*∗∗*^*P* < 0.01 and ^*∗∗∗*^*P* < 0.001 versus control group. ^#^*P* < 0.05, ^##^*P* < 0.01, and ^###^*P* < 0.001 versus LV-shHIF1*α* + ASP group. (c)-(d) Angiogenesis of HCC cells. ^*∗∗∗*^*P* < 0.001 versus control group. ^#^*P* < 0.05, ^##^*P* < 0.01, and ^###^*P* < 0.001 versus LV-shHIF1*α* + ASP group.

To assess the inhibitory effect of ASP combined with HIF-1*α* RNAi on migration and invasion, we treated HCC cells (SK-Hep1 and Hep-3B) with HIF-1*α* RNAi for 24 h (migration) or 48 h (invasion) together with ASP at 10 mg/mL. The results showed that compared with the control group, the migration ability of cells was decreased after treatment with ASP or HIF-1*α* RNAi alone, and the effect was similar in both groups, indicating that ASP or HIF-1*α* RNAi could affect the migration ability of HCC cells. However, the migration ability of cells in the group treated with HIF-1*α* RNAi combined with ASP was further inhibited compared with that of cells treated with ASP or HIF-1*α* RNAi alone (Figures 1(a), 1(b), 2(a), and 2(b)). In terms of invasion ability, cells in the control group had a strong ability to penetrate transwell chambers, whereas the invasion ability of the cells in the group treated with ASP or HIF-1*α* RNAi group was decreased. In addition, cells treated with HIF-1*α* RNAi and ASP were significantly less invasive than those treated with ASP or HIF-1*α* RNAi (Figures 2(a) and 2(b)).

### 3.3. HIF-1*α* RNAi Combined with ASP Inhibited HCC Cell-Induced HUVEC Tube Formation

To analyze the combined effect of ASP and HIF-1*α* RNAi on HUVEC tube formation induced by HCC cells, we conducted a HUVEC-simulated angiogenesis test. HUVECs were cultured on matrix gel and migrated to form a capillary structure with lumen, simulating vascularization. Our results showed that HUVECs cultured in the supernatant of ASP-treated or HIF-1*α* RNAi-treated HCC cells (SK-Hep1 and Hep-3B) presented significantly reduced tubular formation capacity compared with HUVECs cultured in control HCC cell culture supernatant. Consistently, the supernatant of HCC cells undergoing combined treatment with HIF-1*α* RNAi and ASP induced further inhibition, with HUVECs cultured in this supernatant forming less firm capillary and cord structures (Figures 2(c) and 2(d)).

### 3.4. HIF-1*α* RNAi Combined with ASP Inhibited the Expression of HIF-1*α* and VEGF in HCC Cells

**Figure 3:**
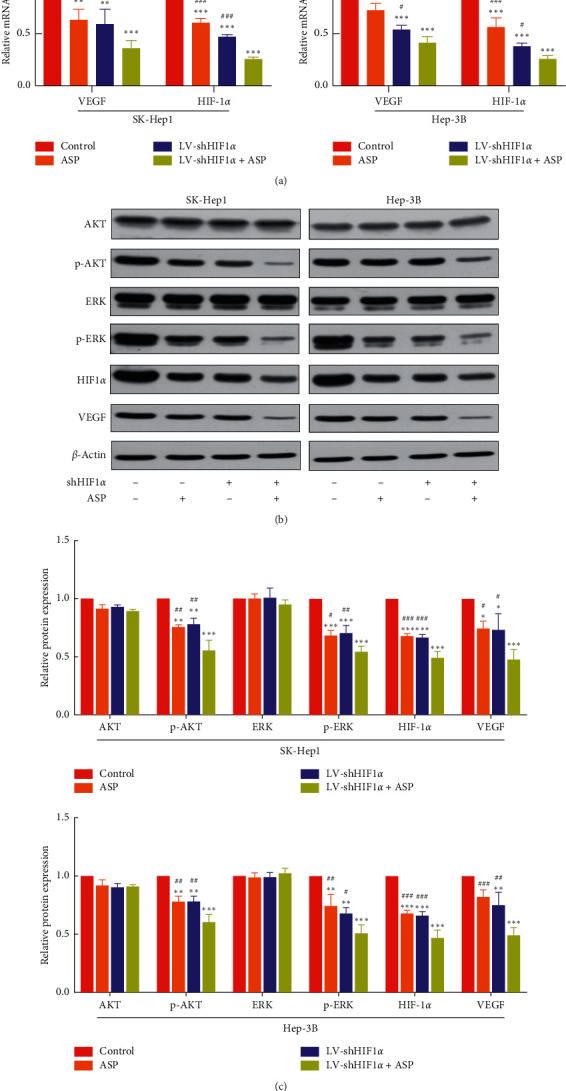
Effect of HIF-1*α* RNAi combined with ASP on the expression of MAPK and PI3K signaling pathway-related proteins. (a) Relative expression of HIF-1*α* and VEGF mRNA analyzed using qPCR. ^*∗∗*^*P* < 0.01 and ^*∗∗∗*^*P* < 0.001 versus control group. ^#^*P* < 0.05, ^##^*P* < 0.01, and ^###^*P* < 0.001 versus LV-shHIF1*α* + ASP group. (b)-(c) Protein levels of HIF-1*α*, VEGF, AKT, p-AKT, ERK, and p-ERK assessed using western blot. ^*∗*^*P* < 0.05, ^*∗∗*^*P* < 0.01, and ^*∗∗∗*^*P* < 0.001 versus control group. ^#^*P* < 0.05, ^##^*P* < 0.01, and ^###^*P* < 0.001 versus LV-shHIF1*α* + ASP group.

To verify whether the mechanism of ASP combined with HIF-1*α* RNAi inhibiting the proliferation, migration, invasion, and angiogenesis of HCC cells was correlated with HIF-1*α* and VEGF levels, the expression of HIF-1*α* and VEGF was assessed using qPCR (Figure 3(a)) and western blotting (Figures 3(b) and 3(c)). The results showed that compared with the control group, the mRNA and protein expression of HIF-1*α* and VEGF in the ASP-treated group or HIF-1*α* RNAi-treated group was downregulated, while that in the HIF-1*α* RNAi and ASP combined treatment group was lower than that in the other two groups.

### 3.5. HIF-1*α* RNAi Combined with ASP Inhibited HIF-1*α* Expression in Human HCC Cells by Regulating the PI3K and MAPK Signaling Pathways

MAPK and PI3K are known to play important roles in regulating HIF-1*α* protein expression. Therefore, inhibition of the MAPK and PI3K signaling pathways may prevent its expression. Previous experiments have confirmed that HIF-1*α* RNAi combined with ASP treatment inhibits the expression of HIF-1*α* and VEGF proteins. To clarify whether this effect was achieved by inhibiting the MAPK and PI3K signaling pathways, we evaluated the protein levels of AKT, p-AKT, ERK, and p-ERK (involved in the MAPK and PI3K signaling pathways) in SK-Hep1 and Hep-3B cells 24 h after treatment with ASP and/or HIF-1*α* RNAi. The results showed that the combined treatment significantly downregulated p-AKT and p-ERK in both cell lines but had no significant effect on the levels of AKT and ERK (Figures 3(b) and 3(c)), suggesting that HIF-1*α* RNAi and ASP regulate the expression of HIF-1*α* in HCC cells by inhibiting the MAPK and PI3K signaling pathways, thereby inhibiting the migration, invasion, and angiogenesis of these cells.

### 3.6. HIF-1*α* RNAi Combined with ASP Inhibited Tumor Growth in a Subcutaneous Xenograft Mouse Model

**Figure 4:**
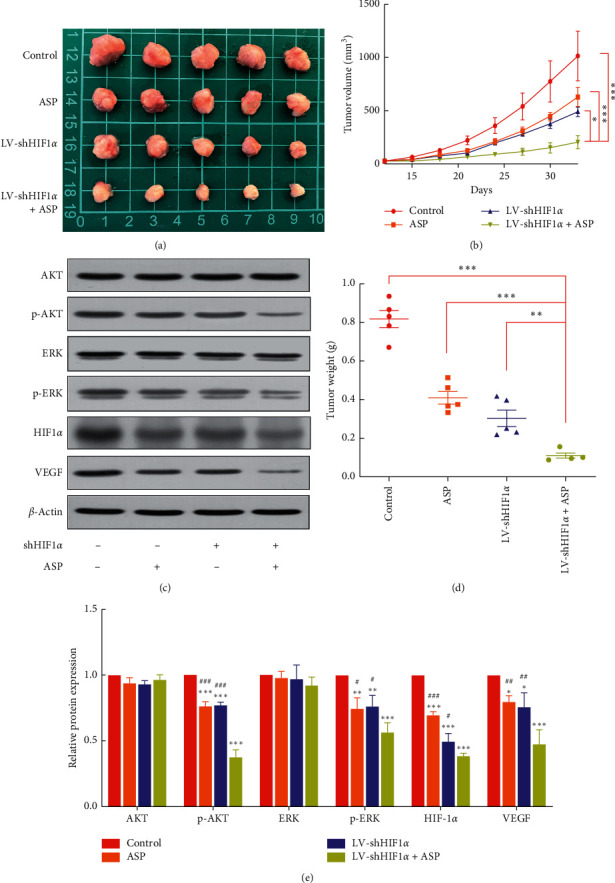
Effect of HIF-1*α* RNAi combined with ASP on HCC growth and angiogenesis in a SK-Hep1 xenograft nude mouse model. (a)-(b), (d) Tumor volume and weight. ^*∗*^*P* < 0.05, ^*∗∗*^*P* < 0.01, and ^*∗∗∗*^*P* < 0.001 versus LV-shHIF1*α* + ASP group. (c), (e) Protein levels of HIF-1*α*, VEGF, AKT, p-AKT, ERK, and p-ERK assessed using western blot. ^*∗*^*P* < 0.05, ^*∗∗*^*P* < 0.01, and ^*∗∗∗*^*P* < 0.001 versus control group. ^#^*P* < 0.05, ^##^*P* < 0.01, and ^###^*P* < 0.001 versus LV-shHIF1*α* + ASP group.

We further investigated the effect of HIF-1*α* RNAi combined with ASP on HCC in vivo. Based on the above-mentioned results, the anticancer effect of HIF-1*α* RNAi combined with ASP was more obvious in SK-Hep1 cells than in Hep-3B cells. Therefore, the former were selected to further study the effect of the combined treatment in a subcutaneous xenograft model using nude mice. SK-Hep1 cells (5 × 10^6^) were subcutaneously inoculated into the armpits of mice to produce tumors. After tumor formation, the ASP and HIF-1*α* RNAi + ASP groups were administered ASP gastrically (100 mg/kg) every day for 3 consecutive weeks. Compared with the control group, the ASP and HIF-1*α* RNAi groups showed inhibited tumor growth and reduced tumor weight. Furthermore, HIF-1*α* RNAi + ASP significantly inhibited tumor growth and reduced tumor weight (Figures 4(a), 4(b) and 4(d)).

### 3.7. HIF-1*α* RNAi Combined with ASP Inhibited Angiogenesis in Subcutaneous HCC Xenografts in Nude Mice

**Figure 5:**
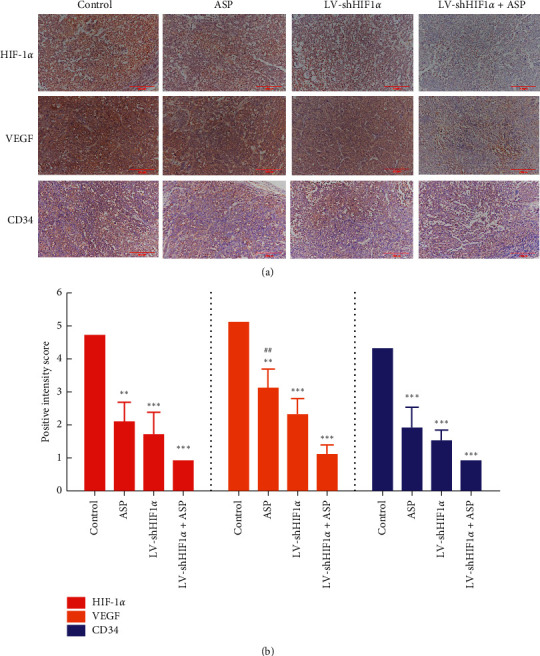
Effect of HIF-1*α* RNAi combined with ASP on the expression of HIF-1*α*, VEGF, and CD34 in subcutaneously transplanted tumors assessed using immunohistochemistry. ^*∗∗*^*P* < 0.01 and ^*∗∗∗*^*P* < 0.001 versus control group. ^##^*P* < 0.01 versus LV-shHIF1*α* + ASP group.

To further clarify the mechanism by which HIF-1*α* RNAi + ASP inhibits the growth of subcutaneously transplanted HCC tumors in nude mice, IHC staining was performed in tumors from all groups (HIF-1*α* RNAi + ASP, HIF-1*α* RNAi, ASP, and control), and the expression of HIF-1*α*, VEGF, and CD34 in tumors was assessed. The results of IHC staining showed that, compared with the HIF-1*α* RNAi or ASP groups, the staining intensity of VEGF and CD34 in tumors from the HIF-1*α* RNAi + ASP group was significantly decreased (Figures 5(a) and 5(b)). These results indicate that inhibition of subcutaneous xenograft growth by HIF-1*α* RNAi + ASP was correlated with the inhibition of angiogenesis in HCC tissues in nude mice.

### 3.8. HIF-1*α* RNAi Combined with ASP Prevented Angiogenesis in Subcutaneous HCC Xenografts in Nude Mice by Inhibiting the MAPK and PI3K Signaling Pathways

Current studies have confirmed that the activation of the MAPK and PI3K signaling pathways promotes the expression of HIF-1*α*, induces the production of downstream VEGF, promotes tumor angiogenesis, and indirectly promotes tumor growth [17, 18]. The above in vitro studies also confirmed that HIF-1*α* RNAi combined with ASP inhibits the expression of proteins related to the MAPK and PI3K signaling pathways in HCC cells. To further verify the mechanism by which HIF-1*α* RNAi combined with ASP inhibits angiogenesis in subcutaneously transplanted HCC in nude mice, the expression of HIF-1*α*, VEGF, AKT, p-AKT, ERK, and p-ERK was assessed using western blotting. The results showed that compared with the HIF-1*α* RNAi or ASP group, protein levels of HIF-1*α* and VEGF in tumors from nude mice transplanted with HIF-1*α* RNAi-treated cells were significantly downregulated (Figures 4(c) and 4(e)). Western blot results also showed that compared with the ASP-treated group or the HIF-1*α* RNAi-treated group, the levels of p-AKT and p-ERK in tumors from the HIF-1*α* RNAi + ASP group were significantly downregulated, but those of AKT and ERK remained the same (Figures 4(c) and 4(e)). These results indicate that HIF-1*α* RNAi + ASP inhibits angiogenesis in nude mice subcutaneously transplanted with HCC, possibly by inhibiting the MAPK and PI3K signaling pathways.

## 4. Discussion

HCC is one of the most common malignant tumors worldwide and a major cause of cancer-related deaths with poor prognosis [19, 20]. Although many studies have focused on the cellular and molecular mechanisms underlying HCC, current therapies are still inadequate, and there is a long way to go before the desired therapeutic effect on HCC can be clinically achieved; thus, more effective therapies need to be developed. Asparagus is a traditional Chinese herbal medicine used in China that contains bioactive compounds such as flavonoids, lignans, steroids, and saponins, which are widely used in the treatment of breast cancer, malignant lymphoma, leukemia, and lung cancer. ASP is one of the main components of aspartame extract, which improves immunity and liver function [21]. In our previous study, we demonstrated that ASP, a Chinese herbal extract, inhibited HIF-1*α*-mediated angiogenesis under hypoxic conditions [16]. Based on this, we silenced the HIF-1*α* gene using RNAi to determine the effect of RNAi technology combined with ASP on cancer angiogenesis and provide an experimental basis for comprehensive clinical anticancer therapy using such combinations.

Hypoxia is the most common microenvironmental feature of many solid tumors, including HCC [22, 23], and is a key factor in the occurrence, prognosis, and metastasis of liver cancer [24]. HIF is a transcription factor that helps cells sense and adapt to changes in oxygen levels [25]. The HIF-1*α*-related signal transduction pathway is one of the most important pathways in HCC angiogenesis and is also the main signaling pathway that induces VEGF [26]. Under normoxic conditions, HIF-1*α* nucleosides are hydroxylated and rapidly degraded through the ubiquitin-proteasome pathway, whereas under hypoxia, HIF-1*α* is no longer degraded, enters the nucleus, and binds to HIF-1*α* protein kinase, thereby activating downstream gene transcription [27]. Furthermore, HIF-1*α*-mediated HCC angiogenesis may be affected by the upstream AKT pathway [28] and AKT in PI3K under the condition of phosphorylation and activation of AKT kinase, further activating the mechanistic target of rapamycin (mTOR). In turn, this stimulates the expression of HIF-1*α*, regulating VEGF expression, with angiogenesis playing a key role in this process. VEGF is an endothelial cell mitogen that promotes the division and migration of vascular endothelial cells and trophoblasts to induce tissue neovascularization. It can also promote the formation of new blood vessels by regulating vascular permeability [29]. Furthermore, VEGF is an important target gene for HIF-1*α* because under hypoxic conditions, HIF-1*α* not only directly promotes its transcription but also promotes its expression by increasing the stability of VEGF mRNA [26].

Tumor angiogenesis is a tumor vascular model independent of endothelial cells, also known as angiogenic mimicry; that is, tumor cells (not vascular endothelial cells) are arranged on the outer surface of the tube wall, and red blood cells are observed in the lumen. Angiogenic mimicry is a microcirculatory system responsible for tumor growth, together with endothelial-dependent blood vessels and mosaic blood vessels [30]. Therefore, inhibiting angiogenesis is a feasible and reliable treatment method for achieving anticancer effects. Our in vivo and in vitro results confirmed that the effect of treatment with HIF-1*α* RNAi combined with ASP was more obvious on HCC angiogenesis than the effects of HIF-1*α* RNAi and ASP treatment and that this combined treatment significantly lowered the p-ERK and p-AKT levels but had little influence on the ERK and AKT levels. This indicates that the combination of ASP and HIF-1*α* RNAi for the treatment of liver cancer may act by inhibiting angiogenesis through the PI3K and MAPK signaling pathways.

In addition to angiogenesis, HIF-1*α* is also involved in the proliferation, apoptosis, invasion, and metastasis of HCC cells. We found that the combination of HIF-1*α* RNAi and ASP significantly inhibited the proliferation, invasion, and migration of HCC cells compared with ASP alone and HIF-1*α* RNAi alone. Previous studies have shown that HIF-1*α* promotes cell proliferation by regulating cyclin A and cyclin D [31]. The hypoxic microenvironment and new blood vessels provide a “hotbed” and “nourishment,” respectively, for HCC metastasis, and cell matrix degradation and epithelial-mesenchymal transition (EMT) lead to poor prognosis of patients with HCC. Moreover, in the process of liver cancer metastasis, HIF-1*α* regulates zinc finger transcription factors to affect the expression of EMT markers, such as E-cadherin, N-cadherin, and vimentin, and promotes invasion and metastasis [32]. How HIF-1*α* and ASP affect HCC cell metastasis will be the focus of our future research.

RNAi is a powerful tool for suppressing the expression of specific genes and has been widely used in targeted cancer therapies. Activated RNAi can achieve specific and powerful gene silencing, providing more possibilities for the clinical treatment of HCC [33, 34]. RNAi can regulate cancer cells within cells to improve the efficiency of cancer treatment. These target genes, which are indispensable for tumor maintenance, have few side effects and low risk, can be knocked out or suppressed by RNAi, blocking the inherent immunosuppression and triggering an immune attack on the tumor. Therefore, cancer is one of the main targets for RNAi-based therapy, as it is highly correlated with gene expression and cell proliferation [35]. Experimental studies aimed at silencing HCC-related oncogenes have shown promising prospects. In particular, using RNAi to inhibit the expression of hepatocyte transformation-related genes is an exciting new approach for the treatment of HCC [36]. Moreover, other therapies combined with gene therapy exert stronger anticancer effects than single-gene therapy [37, 38]. In addition to stronger resistance to HCC proliferation, migration, invasion, and angiogenesis, our combination of gene interference technology targeting the key HCC factor HIF-1*α* with ASP in the treatment of HCC may have profound implications: (1) ASP can improve the efficiency of gene targeting and increase the transfection rate; (2) ASP can ensure that the RNAi technology is locally and directly released, reducing the incidence of adverse reactions to gene therapy; and (3) combined with ASP, gene therapy can alleviate drug resistance, and its therapeutic effect can be enhanced. Notably, the mechanism by which toxicity and drug resistance are reduced deserves further study.

## 5. Conclusions

Overall, our results indicated that ASP combined with HIF-1*α* RNAi inhibited HCC cell growth via the PI3K and MAPK signaling pathways. Thus, we suggest that this combination may be an effective method of integrating Western medicine and traditional Chinese medicine for the treatment of liver cancer, which may provide new ideas for the treatment of various malignant tumors such as HCC.

## Data Availability

All data obtained or analyzed during this study are included within the article.

## Additional Points

*Reporting Checklist.* The authors have completed the ARRIVE reporting checklist.

## Supplementary Materials

Supplementary Materials The ARRIVE Guidelines Checklist.
